# IMproving facial PRosthesis construction with contactlESs Scanning and Digital workflow (IMPRESSeD): study protocol for a feasibility crossover randomised controlled trial of digital versus conventional manufacture of facial prostheses in patients with orbital or nasal facial defects

**DOI:** 10.1186/s40814-023-01351-w

**Published:** 2023-07-03

**Authors:** Rachael Y. Jablonski, Trevor J. Coward, Paul Bartlett, Andrew J. Keeling, Chris Bojke, Sue H. Pavitt, Brian R. Nattress

**Affiliations:** 1 Department of Restorative Dentistry, School of Dentistry, University of Leeds, Leeds, UK; 2 Academic Centre of Reconstructive Science, Faculty of Dentistry, Oral and Craniofacial Sciences, King’s College London, London, UK; 3 Maxillofacial Laboratory, Leeds Dental Institute, Leeds Teaching Hospitals NHS Trust, Leeds, UK; 4 Academic Unit of Health Economics, Leeds Institute of Health Sciences, University of Leeds, Leeds, UK; 5 Dental Translational and Clinical Research Unit, School of Dentistry, University of Leeds, Leeds, UK

**Keywords:** Maxillofacial prosthesis, Head and neck neoplasms, Computer-aided design, Three-dimension printing, Feasibility studies, Cross-over studies, Randomised controlled trial, Qualitative research, Technology assessment, Biomedical

## Abstract

**Background:**

Facial prostheses can have a profound impact on patients’ appearance, function and quality of life. There has been increasing interest in the digital manufacturing of facial prostheses which may offer many benefits to patients and healthcare services compared with conventional manufacturing processes. Most facial prosthesis research has adopted observational study designs with very few randomised controlled trials (RCTs) documented. There is a clear need for a well-designed RCT to compare the clinical and cost-effectiveness of digitally manufactured facial prostheses versus conventionally manufactured facial prostheses. This study protocol describes the planned conduct of a feasibility RCT which aims to address this knowledge gap and determine whether it is feasible to conduct a future definitive RCT.

**Methods:**

The IMPRESSeD study is a multi-centre, 2-arm, crossover, feasibility RCT with early health technology assessment and qualitative research. Up to 30 participants with acquired orbital or nasal defects will be recruited from the Maxillofacial Prosthetic Departments of participating NHS hospitals. All trial participants will receive 2 new facial prostheses manufactured using digital and conventional manufacturing methods. The order of receiving the facial prostheses will be allocated centrally using minimisation. The 2 prostheses will be made in tandem and marked with a colour label to mask the manufacturing method to the participants. Participants will be reviewed 4 weeks following the delivery of the first prosthesis and 4 weeks following the delivery of the second prosthesis. Primary feasibility outcomes include eligibility, recruitment, conversion, and attrition rates. Data will also be collected on patient preference, quality of life and resource use from the healthcare perspective. A qualitative sub-study will evaluate patients’ perception, lived experience and preference of the different manufacturing methods.

**Discussion:**

There is uncertainty regarding the best method of manufacturing facial prostheses in terms of clinical effectiveness, cost-effectiveness and patient acceptability. There is a need for a well-designed RCT to compare digital and conventional manufacturing of facial prostheses to better inform clinical practice. The feasibility study will evaluate key parameters needed to design a definitive trial and will incorporate early health technology assessment and a qualitative sub-study to identify the potential benefits of further research.

**Trial registration:**

ISRCTN ISRCTN10516986). Prospectively registered on 08 June 2021, https://www.isrctn.com/ISRCTN10516986.

## Background

Head and neck cancer (HNC) is the eighth most common cancer in the United Kingdom (UK) [1]. Incidence rates have increased by 34% over the last 3 decades and around 12,400 new cases are diagnosed each year [1]. HNC is commonly diagnosed at a late stage with advanced disease and primary cancer treatment may involve surgery, radiotherapy and/or chemotherapy [1, 2]. Cancer treatment can have numerous consequences due to the complexity of head and neck anatomy and may result in the complete or partial loss of a facial part [3, 4]. The resulting facial defects may be surgically reconstructed or prosthetically rehabilitated using silicone facial prostheses.

A facial prosthesis is a removable replacement for a facial part which may help to improve a patient’s appearance, function, comfort and quality of life [5, 6]. Facial prostheses may be provided for a variety of conditions and conservative estimates suggest over 2000 prostheses are made each year in the UK [7]. Facial prostheses will deteriorate over time, for example through colour change or physical degradation [8, 9]. Furthermore, patients may exhibit seasonal changes to their complexion or physical changes to their soft tissues during healing. Therefore, facial prostheses require regular refurbishment and replacement on a 6- to 24-month basis which presents a long-term impact on both patients and healthcare services [9].

Conventional manufacturing of facial prostheses involves multiple clinical and laboratory procedures including a facial impression, handcrafting a wax pattern and manually converting the wax pattern into a silicone prosthesis [10]. Conventional manufacturing processes can have a variety of limitations from both the patient and healthcare service perspective. For example, large facial impressions can be uncomfortable or claustrophobic for patients and may distort the soft tissues which could impair final prosthetic fit [11–13]. It has been suggested that it will take approximately 9 to 10 h to manufacture a facial prosthesis with the manufacturing costs estimated to be in the region of £1000 to £1500 at one UK hospital [6, 14, 15]. Consequently, conventional manufacturing of facial prostheses is considered to be time, labour and skill intensive [10, 12, 16, 17]. The final result will depend upon many patient and service factors including the maxillofacial prosthetist’s skills and experience [12, 16].

There has been increasing interest in the use of computer-aided design and computer-aided manufacturing (CAD-CAM) technologies for facial prosthesis manufacture in recent years [18]. A systematic review has illustrated that a wide variety of digital techniques have been applied to different stages of facial prosthesis manufacture [19]. These techniques may offer many benefits to patients and healthcare services for example, 3D facial scanning could be a contactless, comfortable and accurate alternative to facial impressions [12, 13]. CAD processes could reduce dependence on the maxillofacial prosthetist’s skills, speed up the prosthesis design stage and enable patients to input through real-time adjustments [16]. Digital skin colour measurements and colourant recipes may help to improve the objectivity and efficiency of colour matching [17, 20, 21]. CAM processes could offer efficiencies in the time and number of stages for production although it is acknowledged that facial prosthesis manufacture has not yet shifted to complete digital fabrication [16, 19, 22–24]. Whilst a broad range of technologies have been introduced to facial prosthesis manufacture, there is currently no single set of standards exclusive to digitally manufactured facial prostheses [19].

A recent systematic review of the outcome measures used in facial prosthesis research found that clinical research in this area was largely restricted to observational studies and only identified 2 previously published randomised controlled trials (RCTs) [25]. Kiat-Amnuay et al. conducted a multicentre crossover RCT which focussed on facial prosthesis materials [26, 27]. During the study, duplicated moulds were used to produce 2 prostheses from silicone and chlorinated polyethylene elastomer materials [26, 27]. Their RCT was terminated early following the recruitment of 42 participants due to recruitment futility resulting from strict eligibility criteria [26]. Abd El Salam et al. conducted a pilot crossover RCT with a small number of participants which compared conventional manufacture of orbital prostheses with a technique involving rapid prototyping [23]. Although the intervention prostheses were designed based on mirroring techniques and manufactured based on the fused deposition modelling of prosthesis replicas, the authors processed the intervention and control prostheses on casts poured from the same impression [23]. Very specific clinical applications of digital technology had been explored as the fit of their intervention prostheses would still be based upon conventional impression processes. Therefore, there remains a need to formally evaluate the clinical application of digital technology more broadly across 3D facial scanning, CAD and CAM.

Furthermore, there has been limited consideration of the health economic impact of facial prostheses interventions [25]. The manufacturing times and costs reported in the literature are often stated without presenting formal evaluations or are based upon the findings of case reports [6, 14, 15]. Studies that capture health-related quality of life, health utility or costs could help generate evidence to support health economic evaluations [25]. Faris et al. evaluated the health utility of different health states with naïve observers and reported visual analogue scale, standard gamble and time trade off derived health utilities for the post-rhinectomy nasal defect, postsurgical reconstruction and post-prosthetic rehabilitation [28]. Ryan et al. conducted a retrospective observational study to compare costs to the hospital associated with auricular reconstruction with either autologous repair or implant retained prostheses [29]. Further studies are required that collect robust data on both the costs and outcomes associated with different methods of facial prosthesis manufacture to inform cost effectiveness analysis.

Consequently, there is a need for a well-designed RCT to compare the clinical and cost-effectiveness of digitally manufactured versus conventionally manufactured facial prostheses. An initial feasibility RCT would provide crucial information about the key parameters needed to plan a definitive trial. Early health technology assessment could support health economic evidence development during the early stages of clinical research and help prioritise future research needs [30]. Furthermore, qualitative research with patients could provide insight into the lived experience of facial prosthesis manufacturing processes and help evaluate the potential application of digital technology from a patient perspective.

## Methods

### Objectives

The aim of the IMPRESSeD study is to determine if it is feasible to conduct a future definitive crossover RCT to evaluate the clinical and cost-effectiveness of digitally manufactured facial prostheses with conventionally manufactured facial prostheses in patients with orbital and nasal facial defects. The primary objective of the study is to assess eligibility, recruitment, conversion and attrition rates.

The secondary objectives are to:

- Investigate the acceptability of the study design and intervention
- Measure key outcome domains relating to preference, health-related quality of life, manufacturing time and costs to synthesise data to inform the sample size of a definitive trial
- Determine the feasibility of the outcome measures as a method to measure the effectiveness of the trial treatments within a definitive trial
- Determine the feasibility of running a multicentre RCT
- Evaluate the feasibility of delivering various components of the intervention within a protocol
- Develop, populate and evaluate an early-stage health economic evaluation:
  - Identify and evaluate the main drivers for cost-effectiveness using available evidence
  - Explore and evaluate model uncertainty using the value of the information framework
  - Determine optimal decision within the health economic framework for making decisions under uncertainty
- Undertake a qualitative sub-study to explore patients’ perception, experience and preference of the 2 different methods of facial prosthesis manufacture

### Trial design and study setting

The IMPRESSeD study is co-designed with Patient and Public Involvement and Engagement (PPIE) contributors who wear facial prostheses and the SMILE AIDER PPIE Forum which ensures the study addresses questions of importance to participants and maximises patient benefits. The study design is a multi-centre, 2-arm, crossover, feasibility RCT with early health technology assessment and qualitative research. All trial participants will receive 2 new facial prostheses which will be manufactured using digital and conventional manufacturing methods.

Participants will be recruited from the Maxillofacial Prosthetic Departments of participating UK National Health Service (NHS) hospitals which includes Leeds Teaching Hospitals NHS Trust and Guy’s and St Thomas’ NHS Foundation Trust. Under usual care at the NHS hospitals, maxillofacial prostheses are provided free of charge to patients through the centrally funded NHS system. Therefore, provision of the conventional prostheses will be attributed to NHS Treatment Costs [31]. Provision of the intervention prostheses will be a Research Cost and will be supported by the research funders, Sponsor and NHS sites [31].

The 2 prostheses will be made in tandem and marked with a colour label to mask participants to the manufacturing method. The order of receiving the intervention will be allocated using minimisation, a widely accepted alternative to randomisation which is effective in minimising imbalance in the treatment groups across key characteristics in RCTs with small sample sizes [32]. Given the variability of the study population, for example, in the extent of facial defect or type of prosthesis being provided, a crossover design will help minimise the risk of confounding as both manufacturing processes are evaluated with the same participant with each participant acting as their own control [33]. A further advantage of crossover study designs is that fewer study participants are required to obtain the same precision in the estimation of intervention effects compared to a standard parallel study design [33].

Following the 4-week follow-up with the first prosthesis, participants will receive the second prosthesis and the first prosthesis will be retained at the site until the final review visit. A washout period was not deemed necessary as it was anticipated that any effects of the prosthesis on health-related quality of life would be temporary whilst the prosthesis was in regular use and hence carry-over effects were not expected to influence outcomes in the second period [33]. A similar crossover design has been used successfully in RCTs of facial prostheses and complete denture manufacturing [23, 26, 27, 34].

### Eligibility criteria

The inclusion criteria comprise patients who have acquired orbital or nasal facial defects and require a replacement facial prosthesis. Patients must also be capable of giving informed consent, aged 16 years or above and available for follow-up. The feasibility study will initially recruit patients with HNC. The Trial Management Group (TMG) will review recruitment and may consider including patients with other acquired conditions as they will be treated within the same services with the same interventions. The exclusion criteria reflect the appropriateness of a crossover design in evaluating interventions in the treatment of stable, chronic conditions [33]. Exclusion criteria therefore include patients who are receiving active cancer therapy, have plans for major reconstructive surgery or have not received a removable facial prosthesis previously. Patients will also be excluded if they have facial defects due to an underlying congenital aetiology, have known hypersensitivity to the materials used in the research, have pre-existing skin conditions that prevent the delivery of a new prosthesis or are unable to give informed consent.

### Interventions

Each participant will have an intervention and control prosthesis made in tandem by maxillofacial prosthetists. The control facial prosthesis will be produced through conventional manufacturing methods (current standard of care) involving a facial impression, handcrafting a wax pattern and converting the wax pattern into a silicone prosthesis. The intervention facial prosthesis will involve 3D facial scanning and CAD-CAM. Both prostheses will be colour matched using the same digital skin colour measurements and colourant recipe and both moulds will be packed with the same silicone. The prostheses will also include the same implant components (where applicable) and duplicate ocular components for orbital prostheses. The ocular components will be manufactured using the same technique (either using hand painting or digital photographic techniques) according to the maxillofacial prosthetist’s usual standard of care [35]. Members of the research team at the University of Leeds will be involved in the digital design of the intervention facial prostheses; however, the maxillofacial prosthetist will be responsible for finishing, quality assuring and delivering the intervention. There are no criteria for discontinuing or modifying the allocated interventions for a trial participant however participants may be withdrawn for appropriate medical reasons and participants are free to withdraw from the study at any time.

The workflow for manufacturing the intervention facial prosthesis has been informed by a review of the literature and tested during a preclinical laboratory study. 3D facial scanning will be undertaken using a structured light scanner (Artec Space Spider; Artec 3D) which will capture the participant’s facial defect and surrounding facial features. Whilst a broad range of facial scanning techniques are available [19, 36], structured light scanning has been shown to have good accuracy and repeatability at digitising facial defects in an in vitro context [37–39]. CAD will involve the use of a 3D Morphable Face Model (Leeds Face Model; University of Leeds) to help design the facial prosthesis in a statistically meaningful way based upon the participant’s other facial features [40–42]. There will be opportunity to further modify the design using landmark fitting techniques where participants share facial photographs from before their cancer treatment or wearing a previously successful prosthesis [43]. Models of the participant’s facial features and positive or negative prosthesis replicas will be printed in an appropriate resin material (such as Model V2 Resin; Formlabs) at a resolution of 50 μm using a stereolithographic desktop 3D printer (Form 3; Formlabs). Direct silicone printing is not yet suitable for printing definitive prostheses due to limitations in the layer thickness [24]. Instead, the prosthesis replicas will be converted to wax and modified by the maxillofacial prosthetist to ensure marginal adaptation and to add thin margins, additional detail and fine textures [23]. Once approved by the maxillofacial prosthetist and participant, the intervention wax pattern will be converted into a silicone material (such as M511 Maxillofacial Silicone; Technovent Ltd) using standard procedures.

### Outcomes

Primary feasibility outcomes include the proportion of patients approached that were eligible (eligibility rate), invited that were successfully recruited (recruitment rate), eligible that consented (conversion rate) and recruited that dropped out of the study (attrition rate).

Secondary feasibility outcomes include:

- Differences in recruitment rates between sites
- Completion rates, missing data, estimates, variances and 95% confidence intervals for the outcome measures relating to preference, health-related quality of life, timing data and costs
- Numbers of adverse events (AEs) and adverse reactions (ARs)
- Factors related to trial delivery, e.g. success of minimisation, fidelity of masking, compliance with trial schedule, acceptability of the intervention and components of the protocol working together

Health economic feasibility outcomes include:

- Cost per Quality Adjusted Life Year (QALY) on current evidence
- Identification of future research needs via Value of Information framework

### Outcomes for the definitive trial

Outcomes for the definitive trial are anticipated to include participant preference, generic health-related quality of life, condition-specific quality of life and costs from the healthcare perspective. The outcome measures have been chosen based upon a systematic review of the facial prosthesis literature and include participant preference, the Short Form-12 (SF-12v2), EQ-5D-5L, the Toronto Outcome Measure for Craniofacial Prosthetics (TOMCP-27) and resource use questionnaires [25]. Table 1 provides a summary of the planned outcomes and outcome measures to be used in the study along with ideal and maximum timepoints for evaluation.

**Table 1:** Timepoints for collection of outcome measures

Outcome	Outcome Measures	Ideal timepoint of evaluation	Maximum timepoint of evaluation
Participant preference	Participant preference for control or intervention prosthesis captured on CRFs	4 weeks after delivery of the second prosthesis	8 weeks following delivery of the second prosthesis
Condition specific quality of life	TOMCP-27	Baseline	Prior to delivery of either prosthesis
		4 weeks after delivery of each prosthesis	8 weeks after delivery of each prosthesis
Generic health-related quality of life	SF-12v2 and EQ-5D-5L	Baseline	Prior to delivery of either prosthesis
		4 weeks after delivery of each prosthesis	8 weeks after delivery of each prosthesis
Costs from the healthcare perspective	Resource use questionnaire	Timing and consumable data collected at each clinical visit or laboratory stage	1 week after each clinical visit or laboratory stage
Participants’ perception, lived-experience and preference of the two methods of making facial prostheses	Semi-structured interviews (qualitative sub-study)	Sampled across the IMPRESSeD study visits or within 12 months of study completion	Broad time frame helpful to identify what is important to patients whilst going through the manufacturing process and when reflecting on processes in retrospect

### Qualitative sub-study

IMPRESSeD study participants will be invited to take part in a qualitative sub-study. Semi-structured interviews will explore patients’ perception, lived experience and preference of the different manufacturing methods. In addition, a stated preference technique called contingent valuation will be used to explore process utility [44]. Participants will state their preferences in a hypothetical scenario and will be asked if all treatment outcomes were equal, would they prefer to have a facial prosthesis made entirely by hand or using digital technology. Participants will be invited to comment on the thoughts and feelings that influenced their decision. Where participants indicate they would prefer a particular manufacturing process, they will be asked to indicate how long they would be willing to wait for this treatment to inform process utility [45].

### Participant timeline

**Fig. 1:**
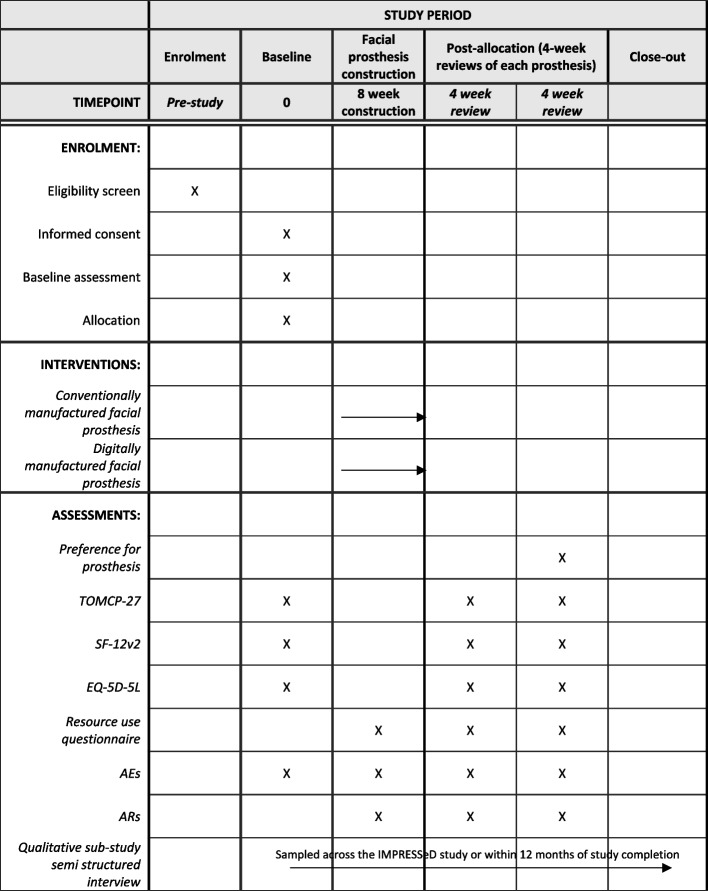
Schedule of enrolment, interventions and assessments [46]

See Fig. 1 for the schedule of enrolment, interventions and assessments.

### Sample size

The feasibility study will estimate treatment effects and variances to inform the sample size of a definitive trial. As there have been very few crossover RCTs in this research area, there was insufficient data available to base the sample size calculation upon the proposed sample size for a definitive trial [47]. The sample size was therefore based upon general recommendations for feasibility studies and a sample size of up to 30 participants was chosen to address the study objectives [48]. It was acknowledged that as the IMPRESSeD study has a crossover design, it would likely have greater power than a parallel-group study and therefore may not need to recruit as many participants as suggested in these general recommendations [49]. Furthermore, consideration was given to the anticipated attrition rates. A dropout rate of up to 33% might be anticipated based upon the findings of a previous crossover RCT comparing facial prosthesis materials [26]. It was noted however that this previous study had a significantly longer follow-up period compared with the IMPRESSeD study (4 months compared to 4 weeks for each prosthesis). The IMPRESSeD study has embedded PPIE into study design and delivery to minimise attrition.

### Recruitment

The multicentre feasibility study will be conducted in at least 2 centres to maximise the likelihood of recruiting the target sample size. The study centres will include Leeds Teaching Hospitals NHS Trust and Guy’s and St Thomas’ NHS Foundation Trust. Both centres provide specialised maxillofacial prosthetic services where patients with facial prostheses attend for routine follow-up visits. Potentially eligible patients will be approached at routine clinical visits or by letter from the clinician to the patient.

### Allocation

**Fig. 2:**
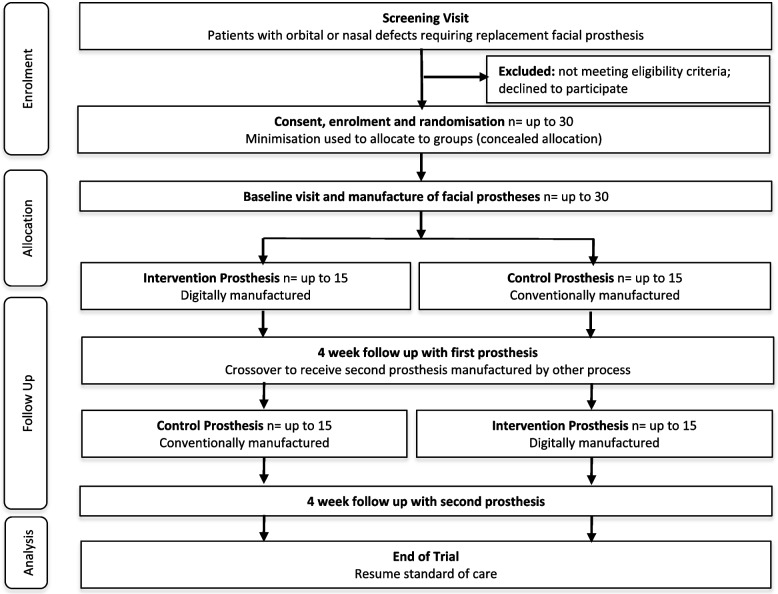
CONSORT flow diagram to illustrate the flow of patients through the feasibility study [50]

In this crossover study, participants will be allocated on the order of receiving the intervention and control prostheses using minimisation [32]. The 2 prostheses will be manufactured in tandem with the sequence of delivery being different between the 2 groups (i.e. group A will receive the intervention followed by the control prosthesis and group B will receive the control followed by the intervention prosthesis) (Fig. 2). A minimisation programme will be set up by a statistician using statistical computing software (RStudio; The R Foundation). The minimisation programme will be designed to achieve balance between the 2 groups for important variables and equal weighting will be given to defect type, retention method and aetiology [32]. It will also incorporate a random element to reduce the predictability of the allocation sequence and each participant will have a 90% chance of being allocated to the treatment group that minimises imbalances [32]. The minimisation programme will be used by the trial coordinator who will generate the treatment allocation of each participant based on the order that they are recruited. Once site team members have recruited a participant, the site will send the randomisation case report form (CRF) to the trial coordinator. The trial coordinator will allocate participants centrally using the minimisation programme, maintain a central log of treatment allocation and send a confirmation email to the site.

### Masking

The intervention and control prostheses will be made in tandem during the same clinical visits. Each pair of prostheses made for each participant will have a similar appearance as they will be manufactured in the same silicone material and using the same colourant recipe. Each prostheses pair will also include the same implant components (where applicable) and duplicate ocular components for orbital prostheses. Trial participants will be masked to the manufacturing method by marking the intervention and control prostheses with a colour label. It will not be possible to mask the operators as the maxillofacial prosthetists will be responsible for prosthesis manufacturing and labelling. The statistician supporting data analysis will be masked to the treatment groups. It is unlikely that participants will need to be unmasked as both prostheses are made in the same material, with the same components and only the method of manufacture will differ.

### Data collection methods

Data will be collected during clinical visits and laboratory stages using CRFs and standardised questionnaires. Data will be collected at baseline and prespecified time points as illustrated in Table 1. Investigators will check CRFs and questionnaires for missing data upon completion. The forms will also be reviewed by the trial coordinator and data queries will be sent to the site for resolution if any data is missing. Investigators may also contact participants over the phone to complete questionnaires to maximise data completeness and maintain data collection time-points. If a participant withdraws from the IMPRESSeD study, any identifiable data will be removed; however, anonymised data will be retained.

Participant preference will be used to indicate whether patients prefer facial prostheses produced by conventional or digital manufacturing methods in a similar way to a study of facial prosthesis retention systems [51]. The TOMCP is a condition-specific quality of life instrument that has been evaluated for reliability and validity during a crossover RCT of facial prosthesis materials [27]. The reduced 27-item, 9-domain version will be used to minimise participant burden [27]. Two generic instruments (SF-12v2 and EQ-5D) have been selected to capture information about health-related quality of life and will also be used to inform the early health technology asssessment [52]. The SF-12v2 is a reliable and valid measure of physical and mental health and the SF-12 has previously been used in studies of auricular prostheses [53–55]. It covers 8 domains which include physical functioning, bodily pain, general health, vitality, social functioning, mental health and role participation with physical and emotional health problems. The EQ-5D is the National Institute for Health and Care Excellence’s preferred measure of health-related quality of life in adults which captures information on 5 dimensions of mobility, self-care, usual activities, pain or discomfort, and anxiety or depression [56]. The responses can be combined into a 5-digit number to describe the participant’s health state. Furthermore, the EQ-5D visual analogue scale captures participants’ self-rated health. The 5-level EQ-5D version will be completed and a cross-walk or further algorithm update will be used to derive utility values [56–58].

Resource use questionnaires will capture the time taken and cost of key consumables using a similar approach to previous intra-oral prosthesis research [59]. The processes to be followed when manufacturing the intervention and control facial prostheses will be outlined in the laboratory working instructions. Maxillofacial prosthetists will be instructed to perform as many adjustments as required to make each wax pattern suitable for the individual participant. Detailed timing and cost data will be captured for each of the clinical visits and laboratory stages at a granular level to provide an understanding of which stages are particularly costly or time-consuming. For example, the manufacture of the intervention wax pattern will be captured as time spent converting the 3D printed replica into wax, time spent adjusting the wax pattern in the laboratory and time spent adapting the wax pattern to the patient clinically. Furthermore, the maxillofacial prosthetists will be asked to document the method used to produce the control wax patterns on the laboratory stages CRF. This will inform the early health technology assessment as the timing data may vary depending upon whether the control wax pattern was sculpted entirely by hand or based on preoperative records, a previous prosthesis, donor records or other methods.

Participants who consent to take part in the qualitative sub-study will be invited to a semi-structured interview to discuss their perception, lived experiences and preference of the 2 different manufacturing methods. Participants will be sampled across the IMPRESSeD study visits or within 12 months of study completion. This sampling method was selected to identify what is important to participants whilst they are going through the manufacturing process and when they reflect on the processes in retrospect. The allocation sequence will not be disclosed to participants prior to the qualitative interview. This will preserve the masking of the study interventions for the main trial, focus the interview on the processes of facial prosthesis manufacture and avoid bias during the stated preference technique.

The interviews will be performed by 2 interviewers and may be held in person, by phone or using a videoconferencing platform according to the participant’s preference. Interviews will typically be conducted by a clinical and a non-clinical researcher. This will provide opportunity to review interview strategies and follow-up questions by involving a second researcher who has not taken part in the manufacture of the facial prostheses. A topic guide has been designed to facilitate an in-depth discussion about patients’ lived experiences of having facial prostheses made, differences and preferences for the 2 different manufacturing methods and the important outcomes of facial prosthesis manufacture. Process utility will be explored using contingent valuation [44]. Interviews will be recorded using an encrypted voice recorder or using the videoconferencing platform and field notes may be collected on an observation guide. Recordings will be transcribed verbatim and pseudonymised. In keeping with the iterative process of qualitative inquiry, the topic guide may be revised following use.

### Data management

All participants will be assigned a unique study identifier which will be used on study documents such as CRFs, questionnaires and transcripts. Participant’s initials and date of birth will also be collected on study forms in the interest of data accuracy. Physical and electronic data will be stored securely, well organised and maintained using appropriate version control. The original CRFs and questionnaires will be sent to the trial coordinator and a copy of the CRFs and questionnaires will be retained in the Investigator Site File as a record of the research undertaken at the site. Data will be entered onto the central trial database and checked for accuracy on a separate occasion. Definitions of important parameters and descriptions of coded or missing data will be maintained.

### Statistical methods

A Consolidated Standards of Reporting Trials (CONSORT) flow diagram will be used to illustrate the flow of patients through the feasibility study (Fig. 2) [50]. Primary feasibility outcomes will be analysed to calculate eligibility, recruitment, conversion and attrition rates. Secondary feasibility outcomes will be analysed using descriptive statistics and statistical comparisons will not be performed between the 2 groups which is in keeping with recommendations for pilot and feasibility studies [60]. The outcome measures will be analysed by exploring completion rates, missing data, point estimates, variances and 95% confidence intervals. There are no planned subgroup, adjusted or interim analyses. Missing data will not be imputed but the variations of some outcomes may be estimated.

The early health technology assessment will adopt a modelling approach instead of a within-trial approach. This will likely involve individual patient-level simulation in which the waiting time of an individual will depend on the volume of patients in the system and the speed of processing. Patient outcomes will be based on Markov-like states with associated costs and utilities [61]. Overall cost-effectiveness will be calculated on a population-averaged basis [62]. The incremental costs and QALYs will be estimated and summary measures such as the Incremental Cost-Effectiveness Ratio and Incremental Net Health/Monetary Benefits will be presented. Uncertainty will be analysed using sensitivity analysis including Probabilistic Sensitivity Analysis which will help quantify the level of confidence in the model output and help prioritise future evidence development [30]. Results will be illustrated on the Cost-Effectiveness Plane and via Cost-Effectiveness Acceptability Curves. The value of information approach will provide a framework to assess whether further research is required, what type is needed and whether the benefits of additional research would exceed the costs [63]. Measures of uncertainty and value of information will be incorporated within the decision-making context to identify optimal decisions on technology adoption or rejection [64]. The early-stage health economic model will be undertaken in accordance with Consolidated Health Economic Evaluation Reporting Standards (CHEERS) recommendations [65].

For the qualitative sub-study, thematic analysis will be used to identify, analyse and report the themes and subthemes within the qualitative data [66]. The exact approach to thematic analysis will be finalised following data collection and familiarisation. However, it is anticipated that the analysis will provide a detailed account of the participants’ experiences of the manufacturing processes, take an inductive approach with the themes linked strongly to the data and will search for semantic themes using the explicit meanings of the data [66]. Thematic analysis will be performed by 2 researchers from different backgrounds and will likely involve a researcher with clinical interests in facial prostheses manufacture and an independent researcher from an alternative background such as psychology. By involving researchers with alternative perspectives, this will aid reflexivity and support the refinement of codes, themes and subthemes.

The lead researcher will listen to the dictations and read the transcripts to become familiar with the depth and breadth of the research data [66]. The researcher will then generate codes to identify features of the data and the codes will be collated and sorted into potential themes and subthemes [66]. Candidate themes and subthemes will be refined in an iterative process to generate a meaningful and coherent thematic map of the data [66]. Feedback from the second researcher will be obtained at key analysis stages for example, after coding the first transcript, after coding all transcripts, and when generating the thematic map. For reporting, the themes will be presented, supported by vivid quotes and described using an analytical approach [66]. Furthermore, findings from the contingent valuation technique may be used to inform the early health technology assessment particularly if participants indicate a preference for a manufacturing method [44, 45].

### Oversight and monitoring

The TMG will comprise the Chief Investigator, Principal Investigators and members of the research team who will meet regularly to discuss study set-up, conduct and analysis. The Independent Advisory Committee (IAC) will oversee the study and will include an independent chair and independent representation from a statistician, a clinical representative and PPIE contributors. The Chief Investigator will attend the IAC meetings which will be held at approximately 6-month intervals. A formal Data Monitoring Committee will not be formed as this is a small, low-risk feasibility study. Both the TMG and IAC will review safety data. The Chief Investigator and Sponsor have the ultimate authority to modify or terminate the feasibility study. The trial coordinator will monitor the study conduct in accordance with the monitoring plan.

### Participant safety

The risks associated with this study are expected to be no higher than the risk of standard care. The intervention is non-invasive and the intended deviation from the current standard of care has been supported by patients’ prioritisation. Potential ARs that may occur during the study may include minor discomfort or soft tissue trauma, biological complications (e.g. candidiasis) or hypersensitivity to materials. The likelihood of these ARs is anticipated to be comparable between the treatment arms. Furthermore, the scanner may use a flashing light which could be a concern to participants with photosensitive epilepsy; however, this risk can be prevented by turning the flashing lights off for the scan when required.

AEs will be documented from the time of informed consent and ARs will be documented from the commencement of trial treatment. All AEs and ARs will be collected throughout the study up until a week after the final follow-up appointment. AEs and ARs may be reported by the participant, discovered by questioning or identified through physical examination. All AEs and ARs will be recorded on the appropriate reporting forms and sent to the trial coordinator for analysis.

### Protocol amendments

This study has been reviewed and a favourable opinion provided by the Leeds East Research Ethics Committee (Reference: 21/YH/0028). Amendments will be prepared and sent to the Sponsor for authorisation. The amendment will be submitted for regulatory approval using the Integrated Research Application System online submission platform. Template emails will be modified and used to notify participating NHS organisations of an amendment. Participants will be notified of relevant amendments and may be asked to sign new versions of Informed Consent Forms when appropriate. The trial registration will be updated accordingly.

### Informed consent

Participants will be provided with information about the study and will be given a minimum of 24 h to decide whether to take part. Informed written consent can only be taken by a member of the research team with delegated responsibility and must be obtained before the participant can undergo any procedures specifically for the purposes of the study. Participants will have the option of allowing information collected about them to be used to support other research in the future and shared anonymously with other researchers.

### Post-study care

As participants will be recruited from secondary care services, once the trial has ended, patients may continue to receive routine care as appropriate at their local NHS hospital according to local policies.

### Dissemination

A final report will be prepared upon completion of the study. The results will be presented at a subject-specific conference and written up for publication within a peer-reviewed journal. Authorship will be based upon International Committee of Medical Journal Editors’ recommendations [67]. This study will also form part of the lead author’s PhD thesis. Plain English summaries will be produced to engage with patient groups.

### Progression criteria

The research team will work with the IAC to determine whether to progress to a definitive trial subject to prespecified progression criteria that have been established based upon the feasibility outcomes. A recommendation for automatic progression would indicate that the trial is considered feasible to deliver in its current format, progression with remedial action would signify that changes to the design of the definitive study would be necessary and halted progression would indicate that a definitive trial is not feasible [68].

The feasibility study recruitment rate and attrition rate are key progression criteria. Progression would be recommended if more than 75% of patients who are approached are recruited and attrition is less than 20%. Alterations to the study design may be recommended if the recruitment rate is between 50 and 75% or attrition is between 20 and 33%. Progression may not be recommended if the recruitment rate is less than 50% or attrition is greater than 33%. These progression criteria will help determine whether the proposed sample size of a definitive trial is achievable.

In the case of non-automatic progression, the research team will consider other feasibility outcomes and the potential impact of mitigating circumstances such as the COVID-19 pandemic to determine whether to progress to a definitive trial. This may include data from the qualitative sub-study relating to patient acceptability of the digital manufacturing workflow and outcomes of the early health technology assessment about the anticipated value of additional research. Furthermore, a review of trial registration databases will be conducted to determine whether any similar studies are in progress or have already been completed.

## Discussion

Despite an increasing interest in the use of digital technology during the manufacture of facial prostheses [18], there is limited information regarding the most clinical and cost-effective methods to prosthetically rehabilitate patients with facial defects. IMPRESSeD has therefore been developed as a feasibility study to explore important parameters needed to design a future definitive RCT. The early health technology assessment will support health economic evidence development during early stages of clinical research, analyse uncertainty and help prioritise future research needs. The qualitative sub-study will offer an important insight into patients’ perception, experience and preferences of the different manufacturing methods.

One of the key challenges anticipated during the IMPRESSeD study is participant recruitment as the study is investigating the rehabilitation of patients with a rare condition. In addition, the feasibility study has experienced delays in set-up due to the COVID-19 pandemic. To mitigate recruitment issues, the study has been co-designed with PPIE contributors to ensure a participant-centred approach to minimise potential burden. The study will be conducted in centres experienced in treating patients with facial defects. Local clinical care teams may approach patients in person or by letter to maximise recruitment. The crossover design means that each participant will act as their own control and therefore fewer participants will be required to obtain the same precision [33]. Furthermore, the design offers a potential benefit to participants as they will be able to keep both prostheses at the end of the study if deemed clinically appropriate by the maxillofacial prosthetist. Recruitment rates will be kept under regular review by the TMG and the eligibility criteria or sample size may be reprofiled in the future.

A wide variety of digital techniques have been used during facial prosthesis manufacture; however, there are currently no clear standards to outline which techniques should be adopted into manufacturing workflows [19, 36, 69, 70]. The digital workflow chosen for this study has been based upon a review of the literature and has been subject to a preclinical laboratory evaluation. The digital workflow integrates the use of 3D Morphable Models and photographic landmark fitting techniques to semi-automate the design of facial prostheses. For all intervention facial prostheses, the CAD-CAM processes will be completed by the same clinician based at the University of Leeds. It is anticipated that the health economic data collected during the study (e.g. timings or material costs) may be influenced by the learning experience that occurs during the early stages of clinical research. Each clinical scenario will come with its own nuances that may require subtly different approaches to CAD (e.g. to incorporate different retention systems or components). Any time spent troubleshooting specific cases will be considered during the early health technology assessment as this would likely improve if the technology was adopted into routine clinical practice.

The participants will be treated by a range of maxillofacial prosthetists due to the amount of clinical and laboratory time involved. All maxillofacial prosthetists will be fully qualified and proficient in providing maxillofacial prostheses and both prostheses will be finished, quality assured and delivered to a similar standard. Maxillofacial prosthetist experience could be a variable that impacts the timing data, for example, if a less experienced clinician takes significantly more time to make a traditional wax pattern compared with the digitally manufactured version. This is one of the key justifications for exploring more standardised approaches using digital techniques in an attempt to simplify time-consuming processes and remove variation among highly experienced maxillofacial prosthetists. At this feasibility study stage, any data gathered will be used to guide the design of a definitive trial and to inform an early health economic model. It is beyond the scope of the study to show a definitive answer for the cost-effectiveness of the 2 treatments or formally explore the impact of these variables. Any findings regarding the potential impact of observable characteristics of the maxillofacial prosthetists (such as years of experience) will be described descriptively to gain an understanding of which variables should be considered when planning a definitive trial.

Further technological developments are to be expected in the future. Both 3D Morphable Models and photographic landmark fitting techniques are active research fields. Therefore, the CAD approach may become more effective over time as the technology becomes better able to reconstruct the shape variations of facial features or recreate the missing facial part more accurately [71]. Furthermore, it is likely that facial prosthesis manufacturing will move closer to complete digital fabrication as direct silicone printing improves further [24]. The IMPRESSeD study will assess the feasibility of conducting a future definitive trial in this research area and start to develop an evidence base during the early stages of clinical research. Opportunities to implement additional technological developments will be considered when planning the future definitive trial.

## Study status

Following confirmation of capacity and capability at the research sites, the Sponsor gave the green light to commence recruitment at Leeds Teaching Hospitals NHS Trust on 1 December 2021 and at Guy’s and St Thomas’ NHS Foundation Trust on 5 May 2022. Recruitment was ongoing at the time of manuscript submission with the anticipated end of recruitment date as 31 January 2023.

## Data Availability

Further information to support the protocol is available from the corresponding author on reasonable request. The datasets generated during and/or analysed during the study will be stored in a publicly available repository (e.g. Research Data Leeds; https://archive.researchdata.leeds.ac.uk/) for a period of 10 years unless there is a legal or ethical reason for destruction. The data will be available upon request after an embargo period. Anonymised participant level data will only be shared for participants who have consented for their data to be used in future research or shared anonymously with other researchers. Data-sharing agreements will be agreed before data is released.

## Abbreviations

AE: Adverse event
AR: Adverse reaction
CAD-CAM: Computer-aided design and computer-aided manufacturing
CHEERS: Consolidated Health Economic Evaluation Reporting Standards
CONSORT: Consolidated Standards of Reporting Trials
CRF: Case report form
EQ-5D-5L: EuroQol instrument to describe and value health with five dimensions and five levels
HNC: Head and neck cancer
IAC: Independent Advisory Committee
IMPRESSeD: IMproving facial PRosthesis construction with contactlESs Scanning and Digital workflow
ISRCTN: International Standard Randomised Controlled Trials Number
NHS: National Health Service
NIHR: National Institute for Health and Care Research
PPIE: Patient and Public Involvement and Engagement
QALY: Quality-adjusted life year
RCT: Randomised controlled trial
SF-12: Short Form 12 Health Survey
SMILE AIDER: Stakeholder Meaningful Involvement & Engagement Aiding Dental Research PPIE Forum
TOMCP: Toronto Outcome Measure for Craniofacial Prosthetics
TMG: Trial Management Group
UK: United Kingdom
